# Anaesthesia for open wrist fracture surgery in adults/elderly

**DOI:** 10.12688/f1000research.13004.1

**Published:** 2017-11-13

**Authors:** Irene Sellbrandt, Metha Brattwall, Margareta Warrén Stomberg, Pether Jildenstål, Jan G. Jakobsson

**Affiliations:** 1 Anaesthesiology and Intensive Care, Sahlgrenska University Hospital, Mölndal, Sweden; 2 The Sahlgrenska Academy Institute of Health and Care Sciences, University of Gothenburg, Gothenburg, Sweden; 3 Department of Anaesthesiology and Intensive Care Medicine, The Sahlgrenska Academy Institute of Health and Care Sciences, Gothenburg University, Gothenburg, Sweden; 4 Department of Anaesthesia & Intensive Care, Institution for Clinical Science, Karolinska Institute, Danderyd University Hospital, Stockholm, Sweden

**Keywords:** open wrist surgery, anaesthetic technique, regional anaesthesia, general anaesthesia, postoperative pain

## Abstract

Anaesthetic technique for open surgery of acute distal for arm fracture in adults/elderly is not well defined. Regional anaesthesia, general anaesthesia or a combined general and regional block may be considered. General anaesthetic technique, the timing and drug/drug combination for the regional block must also be considered. This is a study around published studies assessing anaesthtic technique for wrist surgery. A systematic database search was performed and papers describing the effect of anaesthetic techniques were included.

We found sparse evidence for what anaesthetic technique is optimal for open wrist fracture repair. In total only six studies were found using our inclusion criteria, which all supported the short term, early recovery benefits of regional anaesthesia as part of multi-modal analgesia. More protracted outcomes and putting the type of block into context of quality of recovery and patients’ satisfaction is lacking in the literature. The risk for a pain rebound when the block vanishes should also be acknowledged. Therefore, further high quality studies are warranted concerning the anaesthetic technique for this type of surgery.

## Introduction

Fracture on the upper limb is common, and fracture on the distal forearm, wrist fracture, is one of most common upper limb fractures. Whether the incident increases or declines at least among women is a matter of discussion ^1, 2^. The incidence is increasing among adults and elderly in Sweden ^3^. There are certainly several factors contributing to the increased risk; the fact that individuals are becoming more active at later stages of life and osteoporosis is prevalent in older individuals are both likely to have importance. In addition, there is seasonal variation of distal wrist fracture with high risk during winter months ^4, 5^.

The best treatment of wrist fracture is debated. Data from the Swedish fracture register ^1, 6^ shows that approximately 20% of acute wrist fractures undergo open surgery. In 2009, Smektala *et al.* ^7^ conducted a cohort study around surgical wrist fracture care in Germany. They found that most patients were elderly females, and the predominant fracture management procedure was percutaneous K-wire osteosynthesis (56% of cases), followed by plate osteosynthesis (44%) and more than half of patients had general anaesthesia (55%).

Surgical repair of wrist fracture can be performed under various anaesthetic techniques. Studies assessing anaesthetic technique, whether regional anaesthesia, general anaesthesia or a combined general and regional anaesthetic approach provides the best perioperative care are not well defined. Currently, there is no firm evidence to support a best technique and no clear consensus guidance is available.

The aim of this study was to identify the currently available data regarding anaesthetic technique, general, regional or combined general and regional anaesthesia, and postoperative pain course for open surgical repair of wrist fracture in adults and elderly.

## Methods

### Search strategy

A systematic database search was performed on PubMed, Scopus and Cochrane databases, searching for articles in the period from 2007 to October 2017 (the last 10 year period), to identify studies regarding the impact of anaesthesia method on pain and long term outcomes after open radius fracture repair. Quantitative as well as qualitative English language studies limited to adults were searched.

The search strategy used the following text words or Medical Subject Headings (MeSH): “Radius fractures, Wrist injuries, Surgery, Anaesthesia, Regional anaesthesia, Pain, Postoperative, Clinical outcome”, combined search for the last 10 years; (radius fractures OR wrist injuries OR wrist fractures) AND anesthesia. The result was further analysed by two of the authors and secondary papers searched from the initial studies retrieved.

### Inclusion and exclusion criteria

Observational studies and case series were excluded, as it was considered that these could be associated with major bias. Study selection was based on an initial screen of titles and abstracts, and then reading the final identified articles. Papers with at least two different techniques assessing clinical peroperative effects were included.

Selection of papers and subsequent extraction was performed independently by two of the authors (MWS and JGJ). Consensus was reached for final article inclusion and data extraction through discussion between all authors.

### Data extraction

A standard data extraction matrix was conducted manually, to gather study information including: references/year of publication/author, anaesthesia method, postoperative pain before and after discharge from hospital.

## Results

**Figure 1.**
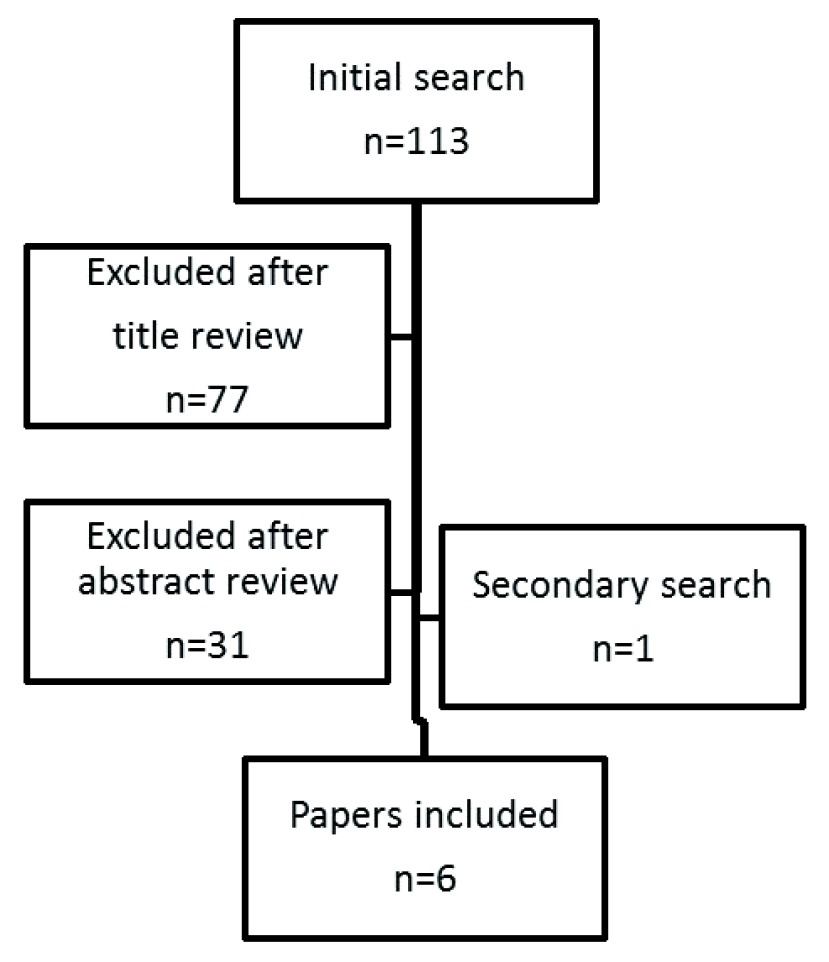
Flowchart showing the literature search results.

Figure 1 describes the search results from the literature.

In total, 6 studies, 4 prospective randomised and 2 retrospective studies of various designs, were identified, which altogether included 619 participants (prospective, n=238; retrospective, n=381) (see Table 1).

**Table 1.** Studies included concerning anaesthesia technique for open wrist fracture surgery in adults/elderly. GA, general anaesthesia; RA, regional anaesthesia; IRA, intravenous regional anaesthesia (Bier block).

References	Design	Number of patients	Anaesthesia method	Postop pain before discharge	Postop pain after discharge
Hadzic *et al,* Anesthesiology 2004	Prospective randomised	50 patients Aged 18–65 years	RA: Infraclavicular plexus block + propofol sedation (chloroprocaine) GA: propofol/desflurane + local infiltration	Assessed until discharge RA less pain and no need for rescue, earlier discharge	No difference in pain or pain medication at 24 and 48 hours, the block group of patients had less pain and pain medication at 72 hours
Egol *et al.*, J Orthop Trauma 2012	Retrospective	187 patients	RA (n=65): Infra clavicular plexus block (LA not presented) GA (n=122): not further presented	Not assessed	3, 6 and 12 month follow up showed better pain and functional course for RA group
Sunderland *et al.*, Reg Anesth Pain Med 2016	Retrospective	195 patients	RA (n=118): Supra or infra clavicular block (LA not presented) GA (n=77): not further presented	GA more early pain before discharge and unplanned health care resource utilization due to pain	RA more self-reported severe pain and - higher unplanned visits up to 48 hours
Galos *et al.*, Clin Orthop Relat Res 2016	Prospective randomised	36 patients	RA (n=18): Infraclavicular plexus block (combined lidocaine 20 mg/ml with adrenaline and bupivacaine 2.5 mg/ml) GA (n=18): not further presented	GA more early pain before discharge	IRA increase in pain 6–24h postop, no difference 48, 72 and at 2 weeks
O’Neil *et al.*, Am J Orthop 2017	Prospective randomised	98 patients	RA (n=53): Single shot supraclavicular block (ropivacaine + dexamethasone) GA (n=45): not further presented	Not specified	Opioid consumption was equivalent for general and regional anaesthesia up to 2 weeks after surgery. Mean duration of opioid use was 4.8 days with a significant trend toward less consumption with increasing age
Holmberg *et al.*, Anaesthesia 2017	Prospective randomised	52 patients	All patents received GA (TIVA) RA pre- or post-op n=26 each: Infraclavicular plexus block (ropivacaine 7.5 mg/ml)	Postoperative pain scores were higher and more patients required rescue analgesia during the first 4 h after surgery in the postoperative block group	Preoperative block showed a better pain course. The risk for rebound was commented

Overall, it could be seen that there is little information around anaesthetic techniques used, and base pain medications is sparsely addressed. The focus has been on early and intermediate pain and opioid consumption. Pain was assessed with different scales in each study.

### Prospective randomised studies

**Hadzic *et al.*** ^8^ performed a study in 2004 comparing general anaesthesia to infraclavicular plexus block with short-acting local anaesthesia for hand and wrist surgery. They included 52 patients; however 2 were excluded from analysis, resulting in 50 patients; 25 each in general and regional anaesthesia were evaluated. The focus was on early recovery during the stay in hospital, but patients were also called on the telephone once daily for days 1, 2 and 3 and interviewed about pain felt. In addition, 2 weeks later the patients were asked about satisfaction and willingness to have the same anaesthesia. They found that the block group needed approximately 5 minutes longer time for preparation prior to surgery, but more patients in the block group were eligible for early recovery room bypass. The block group also experienced less pain, nausea, sore throat and fatigue. The block group were eligible for discharge at a significantly faster rate that the anaesthesia group. Pain at follow-up did not differ at day 1 and 3 between the two groups, but was found in favour, lower pain, for block group of patient at day 3. Satisfaction with anaesthesia at 2 weeks was found to be the same, but willingness to have same anaesthesia favoured the block technique.

**Galos *et al.*** ^9^ randomized patients to general anaesthesia (n=18) or plexus block (n=18) and followed postoperative pain 2, 4, 6, 12, 24, 48 and 72 hours after surgery. They found that the general anaesthesia group had more pain 2 hours postoperatively, but regional anaesthesia experienced more pain 12 and 24 hours after surgery, termed “rebound pain”, as compared to the general anaesthesia patients. There were no differences in pain ratings at 48 and 72 hours or at 2 weeks. Follow-up of function at 2 and 6 weeks did not show any differences.

**O’ Neil *et al.*** ^10^ studied 98 patients, 45 receiving general anaesthesia and 53 regional anaesthesia, a single shot peripheral block, for preoperative management. They had a questionnaire follow-up at a 2-week follow-up visit where the amount of pain medication used was requested. They did not find any difference in opioid need between general and regional anaesthesia groups. Opioid need decreased, however, with age and increased in relation to the severity of the fracture.

**Holmberg *et al.*** ^11^ studied 52 patients with fractured radius. The study compared pre- and post-operative block, in both groups combined with general anaesthesia total intravenous anaesthesia (TIVA). The patients were randomized to a preoperative (n=25) or a postoperative (n=26) infraclavicular plexus block. Time to the first rescue analgesic, as well as pain score evaluation, was at 1, 2, 4, 8 and 24 hours after surgery. Patients were further called for phone interview on day 7 and 6 months postoperatively, and asked about pain, rescue analgesia daily function and side effects. The time for needing first rescue opioid analgesic was significantly longer for the pre-operative block group (544 vs 343 minutes). There was also a significantly lower mean VAS pain score at 30 minutes, 1, 2 and 4 hours. Pain was, however, not different after 4 hours and beyond. At day 7 more patients in the postoperative block group needed oral paracetamol as compared to the preoperative group; however, the total opioid need was the same. During the first postoperative night, most patients from both groups experienced severe pain. Follow-up at 6 months showed no difference in pain; median pain score in the pre-operative group was 2 as compared to the postoperative group median of 1.

### Retrospective studies

**Egol *et al.*** ^12^ published a retrospective register study in 2012 assessing pain after wrist surgery. Included in the analysis were 122 patients that had surgery under general anaesthesia and 65 under a regional block. Patients were followed up for analysis at 3, 6 and 12 months after surgery; pain and function was assessed. At 3 and 6 months, patient that had regional anaesthesia showed significantly less pain and better function; however, at 12 months groups were equal in pain assessment, and function measured by the test were similar and normalised, back to normal range.

**Sunderland *et al.*** ^13^ conducted a retrospective review, for a retrospective quality improvement project, to assess the need for unplanned medical visits caused by pain in the first 48 hours after wrist surgery and the impact of anaesthetic technique, general anaesthesia or regional block. All 77 patients who had general and 118 patients who had regional anaesthesia were reached for follow-up interview within 6 weeks, which posed the question “ *In the first 24 to 48 hours after surgery, did you need to seek medical attention for pain?*”. The block group was found to have significantly more unplanned visits (20% vs 5%), and self-reported severe pain was also more common in this group of patients (41% vs 10%), as compared to the general anaesthesia group of patients. Patients were also asked about persistent post-operative pain and if they had been diagnosed with a “complex regional pain syndrome” (CRPS). No difference in development of chronic pain or diagnosis of CRPS was found between the two groups.

## Discussion

Most wrist fractures in adults and the elderly can be managed by close reposition. However, certain fractures require open reposition and fixation at initial examination, and others fail close reposition and fixation therapy and thus require an open procedure. Wrist surgery is not uncommonly performed as day or over-night stay surgery and is common in elderly patients. Thus, it is of importance to consider various outcomes when comparing anaesthetic techniques used in these procedures and on these patients.

We found only six papers explicitly addressing anaesthetic technique for open acute distal arm surgery. The pharmacological effect of blocking nociceptive influx is clear; preoperative block seems better than post-surgery block. The effect beyond the early period and duration of the pharmacological blockade was more diverse between studies, and it seems that the risk for “rebound” pain should be acknowledged.

The benefits of prevention of pain and the concept of multi modal analgesia have been known for decades ^14^. The importance of multi-modal analgesia is well-accepted, and today this standard of care is especially important in ambulatory surgery ^15^. The application of local anaesthetics in the wound area, site for incision, or as a peripheral block, is a basic part of the multi-modal analgesia concept. The effects of peripheral block on early pain, reducing the need for opioid analgesics are well-known ^16^. The effects beyond the early period and the duration of the pharmacological local block is less well documented ^17^. There are several options to improve the duration by the addition of adjuncts e.g. alf-2-blocking agents, such as clonidine or dexmedetomidine; however, the prolongation of the block resolution by liposomal bupivacaine preparation needs further studies ^18^.

Hadzic *et al.* used propofol followed by desflurane and Holberg *et al.* describes general anaesthesia as TIVA for all patients, the general anaesthetic techniques used were not further addressed. Awakening/emergence from anaesthesia is faster when inhaled anaesthesia has been used, shown by many previous studies ^19, 20^. The benefits of the use of nitrous oxide to quicken emergence is also well documented ^21, 22^. The impact of low blood gas solubility on awakening is also shown with xenon as the main anaesthetic ^23^. Xenon does promote stable intraoperative haemodynamic and rapid recovery, as compared to traditional halogenated inhaled anaesthetics ^24^. The classic study by Apfel *et al.* verified the importance of multi-modal PostOperative Nausea and Vomiting - PONV prophylaxis ^25^. Appropriate risk based PONV prophylaxis should be administered reducing its occurrence ^26^. The benefits of propofol-based anaesthesia to reduce early PONV is well known ^27^.

From the six studies identified, the blocks were done with different techniques and solutions. The studies by Hadzic *et al.*, Egol *et al.*, Galos *et al.* and Holmberg *et al.* used sole infraclavicular block technique, while Sunderland *et al.* used mixed infra- and supraclavicular block technique and O’Neil *et al.* used sole supraclavicular blocks. The local anaesthetics used differed as well: Hadzic *et al,* chloroprocaine; Galos *et al,* mixed lidocaine and bupivacaine; O’Neil *et al.*, ropivacaine and dexamethasone; Holmberg, ropivacaine. Adding adjuncts to increase the analgesic block duration is of interest, but generally is off-label use, and there is no firm consensus about what drug and concentration should be used ^**xiii**^. There is a recent review suggesting dexmedetomidine is superior to clonidine as adjunct for supraclavicular block, but there is also a risk of side effects ^28^.

We did not address anaesthesia/analgesia/sedation for closed reduction; there is a Cochrane review around anaesthesia for the close reposition from 2002 ^29^. Pain associated with the performance of local anaesthetic blockade was not evaluated in this study but may be further taken into consideration in the patient´s participation in anaesthetic decision.

It is obvious that further high quality studies are warranted; studies assessing not only early but more protracted outcome variables. For example, effect on logistics, theatre time, patients eligible for fast-track, bypassing recovery area and time to discharge should all be included. Effects beyond discharge need to also be assessed in a standardised fashion, pain and analgesic requirement at least up to a week post-surgery and quality of recovery should be assessed by a standardised tool. There are today at least two well-accepted tools for the assessment of quality of recovery, the PostopQRS and the Quality of Recovery scale ^30^. The long-term outcome and the risk for chronic pain is dependent on several factors and the impact on anaesthetic technique may not have major impact. Long term outcomes are of importance, but the impact on training, rehabilitation surgical technique and even osteoporosis must be acknowledged when assessing long-term results. Similarly, considerations must be taken that wrist fracture may initiate complex regional pain syndrome type I. For instance, severe fractures and women are at high risk for development this syndrome after surgical treatment of distal radius fractures (Roh *et al.*., 2014) ^31^.

In conclusion, there is sparse evidence for what anaesthetic technique is superior for open wrist repair. The short term, early recovery benefits of regional anaesthesia as part of multi-modal analgesia is well documented; however, more protracted outcomes and putting the type of block into context of quality of recovery and patients’ satisfaction is lacking. The risk for pain rebound when the block finishes should also be acknowledged. Further high quality studies are warranted.

## Notes

^1^ https://stratum.registercentrum.se/#!page?id=1094
