# Supplementary material for: Anaesthesia for open wrist fracture surgery in adults/elderly
Source: F1000Res. 2017 Nov 13;6:1996. [Version 1] doi: 10.12688/f1000research.13004.1 (PMC5770994; doi:10.12688/f1000research.13004.1)
Supplement: Peer review history

# Peer review: Referee response for version 1

**DOI:** 10.5256/f1000research.14100.r29364

**Published:** 2018-01-16

**Authors:** Colin F. Royse

**Affiliations:** 1 Department of Surgery, University of Melbourne, Melbourne, Vic, Australia

The authors have conducted a systematic review to identify if regional versus general anaesthesia is superior for post-operative pain for distal wrist fractures. They correctly show that there is an evidence gap in the literature, and cannot provide recommendation from their data. The study is well conducted, but the contributing trials are all small and have sufficient heterogeneity that it is simply not possible to draw any conclusions, other than more trials need to be done.

I have read this submission. I believe that I have an appropriate level of expertise to confirm that it is of an acceptable scientific standard.

# Peer review: Referee response for version 1

**DOI:** 10.5256/f1000research.14100.r28244

**Published:** 2017-12-11

**Authors:** Jan H. Eshuis

**Affiliations:** 1 Department of Anesthesiology, Academic Medical Center, University of Amsterdam, Amsterdam, Netherlands

The article’s title suggests focusing on the elderly but the studies mentioned do not refer especially to the elderly. Although the authors are right in saying that wrist fractures are more common in the elderly it is not clear in the studies mentioned; I suggest to remove “elderly” from the title. In the text it is rightly mentioned.

 It is interesting that the article shows a relative lack of information in the literature on this specific wrist fracture treatment. I agree with all the conclusions drawn by the authors about desired further studies; important that is noted that rebound pain in the aftercare phase is higher in the regional group than the multimodal group of patients; this provides us an important “mirror” to our daily practice!

I have read this submission. I believe that I have an appropriate level of expertise to confirm that it is of an acceptable scientific standard.
